# Direct and indirect associations of experience of racial discrimination, dietary patterns and obesity in adults from southern Brazil

**DOI:** 10.1017/S1368980024000338

**Published:** 2024-02-01

**Authors:** Marcos Fanton, Ylana Elias Rodrigues, Ilaine Schuch, Caroline Marques de Lima Cunha, Marcos Pascoal Pattussi, Raquel Canuto

**Affiliations:** 1 Postgraduate Program in Philosophy. Universidade Federal de Santa Maria. Santa Maria, RS, Brazil; 2 Postgraduate Program in Nutrition Sciences. Universidade Federal de Ciências da Saúde de Porto Alegre, Porto Alegre, RS, Brazil; 3 Postgraduate Program in Food, Nutrition and Health, Faculty of Medicine, Universidade Federal do Rio Grande do Sul. Porto Alegre, RS, Brazil; 4 Hospital de Clínicas de Porto Alegre (HCPA), Porto Alegre, RS, Brazil; 5 Postgraduate Program in Collective Health, Universidade do Vale do Rio dos Sinos, São Leopoldo, RS, Brazil

**Keywords:** Obesity, Abdominal obesity, Dietary patterns, Racial discrimination, Racism

## Abstract

**Objective:**

To analyse the direct and indirect associations of experience of racial discrimination on dietary patterns (DP), obesity and abdominal obesity.

**Design:**

This is a cross-sectional population-based study. The main exposure was self-reported experiences of racial discrimination (Experiences of Discrimination scale). The mediator variables were the DP: healthy, Brazilian traditional, sugar and carbohydrates, and fast food. The outcomes were obesity (BMI ≥ 30 kg/m^2^) and abdominal obesity (waist circumference ≥ 88 cm for women; ≥ 102 cm for men). Structural equation modelling was applied.

**Setting:**

Porto Alegre, Brazil.

**Participants:**

Totally, 400 adults aged between 20 and 70 years were participated.

**Results:**

The mean age of participants was 47·2 years (s d = 13·9), and 75 % were women. Experiencing racial discrimination had a positive direct effect on obesity (healthy DP: *β* = 0·153, *P* < 0·05; Brazilian DP: *β* = 0·156, *P* < 0·05; sugar and carbohydrates DP: *β* = 0·156, *P* < 0·05; and fast-food DP: *β* = 0·153, *P* < 0·05) and abdominal obesity (healthy DP: *β* = 0·206, *P* < 0·01; Brazilian DP: *β* = 0·210, *P* < 0·01; sugar and carbohydrates DP: *β* = 0·204, *P* < 0·01; and fast-food DP: *β* = 0·204, *P* < 0·01). The experience of racial discrimination did not have a direct effect on DP, nor did it exert an indirect effect on obesity and abdominal obesity through any DP.

**Conclusions:**

A higher experience of racial discrimination is associated with obesity and abdominal obesity, independent of diet.

Racism and racial discrimination against Afro-Brazilians remain a major social and political problem in Brazil. Brazil was not only the last country to abolish slavery but also the one which received the most enslaved Africans compulsory in its territory (it is estimated a population of 4·8 million Africans between 1550 and 1862)^(1)^. The historical legacy of slavery is still seen in a broad range of persistent inequities. Governmental statistics show that Brown and Black Brazilians (which amount to 46·8 % and 9·4 % of the population, respectively) usually have lower income, schooling degree, formal employment, Internet access and political representation, and higher rates of incarceration, deaths due to homicide, law enforcement and early mortality^(2,3)^.

As a social and political determinant of health, racial discrimination is a multidimensional and complex phenomenon. It is often characterised as a ‘racialised social system’ based on practices, mechanisms, values, beliefs, and behaviours that reproduce racial domination on a social group and give privilege and power to a dominant group through racial designations^(4,5)^. This unfair and oppressive treatment of members of a particular social group based on ethnic or racial designations can occur at different levels, from an internalised one to the interpersonal, structural and/or systemic levels^(6,7)^.

Two main methodological approaches have been employed to assess the health implications of discrimination for individuals across diverse racial groups. The predominant approach involves investigating the association between the race variable and health outcomes. However, there is a growing trend in evaluating the effects of discrimination on health-related outcomes using scales measuring experiences of discrimination. These scales are designed to query respondents about their experiences of racial discrimination, enabling the examination of the relationship between discrimination and adverse health outcomes^(8,9)^. Self-reported racial discrimination has been widely adopted as an instrument to measure and assess the impact of racial discrimination on health outcomes^(8,10,11)^. As a daily and prolonged phenomenon, interpersonal racial discrimination harms people’s health as a social stressor, triggering physiological, psychological and behavioural responses to it^(12)^. When these experiences are accumulated during one’s lifetime, they can harm one’s mental and physical health, leading to non-communicable chronic diseases, such as obesity^(12-14)^.

In this sense, previous longitudinal studies have investigated the association between perceived racial discrimination and anthropometric outcomes, demonstrating an association with weight increase, but not with waist circumference increase^(12,15)^. This association seems to be partially explained by the modification of eating behaviours due to stress. A systematic review demonstrated that individuals who endure day-to-day racial discrimination exhibit poor eating behaviours, such as emotional eating, binge eating, and loss of control when eating, and consume unhealthy diets, such as excessive consumption of sweets and fats and lower consumption of fruits and vegetables, due to this kind of stress^(10)^. Another systematic review on the impact of psychological factors (stress, anxiety, depression and discrimination) on emotional eating and weight among American Black women suggested that negatively perceived stress may be predictive of emotional eating, and negative emotions influence overeating among women with overweight and obesity^(16)^.

An alternative route to explain this association lies in the direct link between chronic stress, stemming from experiences of discrimination and heightened body adiposity. The cascade begins with chronic stress triggering the activation of the hypothalamus–pituitary–adrenal axis, subsequently inducing an inflammatory process mediated by glucocorticoid hormones. This inflammatory response, in turn, contributes to increased fat retention and weight gain^(17,18)^.

In Brazil, a cross-sectional analysis demonstrated that Brazilian Black women are more likely to have obesity than White women^(19)^, and a longitudinal analysis found a positive association between racial discrimination and obesity^(20)^. Studies that measure the association of this particular outcome with perceived racial discrimination, and not only with race, skin colour and/or ethnicity, are very scarce in Brazil, though^(14,20)^.

The association between racial markers and food consumption has received even less attention. A review that included data from the largest Brazilian population surveys identified the association between socio-economic status and food consumption have highlighted that only five of the twenty-four studies included colour/race in the analyses. These studies found that the consumption of fruits and vegetables was higher among Whites. On the other hand, cardiovascular risk markers foods were associated with Brown and Black race/colour. No study evaluating the association of racial discrimination with food consumption or eating behaviours was found^(21)^.

Despite the growing evidence of the association between perceived racial discrimination with eating behaviours and obesity, the causal pathways of this relationship remain relatively underexplored and the mediating role of food consumption is not frequently investigated. In addition, as far as we know, only one study evaluated the association of experiences of racial discrimination with obesity and none with food consumption in Brazil. How racial discrimination affects eating behaviour and obesity within different ethnic, cultural, social and economic contexts is paramount for tackling social and political determinants of health beyond the Global North.

Thus, this study aimed to analyse the direct and indirect associations between self-reported experiences of racial discrimination with dietary patterns (DP) and obesity and abdominal obesity in a sample from southern Brazil. The study hypotheses (H) were: (H1) Experience of racial discrimination has a positive direct effect on obesity and abdominal obesity; (H2) Experience of racial discrimination had a positive direct effect on unhealthy DP (sugar and carbohydrates DP and fast-food DP); (H3) Experience of racial discrimination had an inversely direct effect on healthy DP (healthy DP and Brazilian traditional DP); (H4) Experience of racial discrimination had a positive indirect effect on obesity, and on abdominal obesity that was mediated by higher intake of unhealthy DP; and (H5) Experience of racial discrimination has a positive indirect effect on obesity and abdominal obesity that can be attenuated by higher intake of healthy DP.

## Methods

It is a cross-sectional population-based study with a sample of men and women adult individuals aged between 20 and 70 years, who live in the coverage area of a primary healthcare service located in the central area of the City of Porto Alegre, capital of the Rio Grande do Sul State, Brazil. The central area of Porto Alegre has about 260 000 residents who are attended by three primary healthcare centres covering approximately 12 000 households.

The analysis is part of a larger epidemiological study titled ‘Social and Environmental Determinants of Food and Nutrition: An Ecosocial Approach’. This comprehensive study was devised to assess how ecosocial epidemiology can serve as a basis for exploring the social and environmental factors influencing food and nutrition, as well as their associated disparities, within an adult population residing in the central area of a major city in southern Brazil^(22,23)^. The sample size was initially determined to investigate the relationship between income and obesity. The software Epi Info version 7 (Centers for Disease Control and Prevention) was used. The following parameters were adopted for sample size calculation: 95 % confidence level, 80 % statistical power, 1·35 relative risk, unexposed ratio: exposed 1:2, overweight prevalence among the unexposed 43 % and exposed 58 %. A sample of 419 individuals was estimated^(22)^. For this specific study, Kline’s recommendation stipulated a minimum of 200 cases for conducting structural equation modeling analysis^(24)^.

Individuals aged between 20 and 70 years of both genders were eligible for inclusion in the study. Those who had any physical or mental limitations that prevented data acquisition and pregnant women were not included.

A stratified sampling method was adopted. The sample was divided into two parts to guarantee the different socio-economic strata intended in the study. In the lower-income areas with only 250 families, all eligible participants were invited to participate in this study (census sampling); the 201 participants who agreed to participate were included (refusal rate:16 %). In the higher-income areas, the same number of individuals was included to maintain sample proportionality. A random sampling procedure was used to select the primary sampling unit (households) in these areas (refusal rate, 22 %). Only one individual per household was included; when more than one person in the household met the inclusion criteria, one was selected randomly. An effort was made to alternate the gender of the participants for each household included, that is, whenever a woman was included, an attempt was made to include a man in the next household, and vice versa. Data were collected through face-to-face interviews conducted between October 2018 and June 2019.

This study was conducted according to the guidelines laid down in the Declaration of Helsinki and all procedures involving human subjects. Written informed consent was obtained from all participants.

### Experiences of discrimination: exposure variable

The experiences of discrimination (EOD) is an eighteen-item self-report instrument used to measure experiences of discrimination based on race/ethnicity or colour in population health research^(25)^. The version validated among the Brazilian population has thirteen items and comprises two dimensions of the scale: ‘experiences of discrimination’ (nine items) and ‘concern about discrimination’ (four items).^(26)^. The dimension ‘experiences of discrimination’ conceptualised differential or unfair treatment of individuals based on self-identified race, ethnicity or colour. It contains nine items with questions about race/colour discrimination in the following situations: attending school, seeking a job, at work, buying a house, getting medical care, being in a store or restaurant, getting credit, being on the street or in public settings, encountering the police, or being in the courts. Response choices for each situation were never, once, twice or three times, and four or more times. In this study, we only used this first dimension. Each question was dichotomised (never/ =< once time) following the literature recommendation^(25)^. The combined responses to these questions were then aggregated to create a score, ranging from 0 to 8, which was employed in the descriptive analyses. Additionally, for the multivariable analyses, one latent variable was derived through confirmatory factor analysis.

### Dietary patterns: mediator variables

The DP analysed in this study were identified and described in a previous study^(23)^. In brief, food consumption was determined with a validated qualitative FFQ comprising eighty-five food items. Respondents reported on all food items consumed in the past year, recorded in the number of days per week, month or year. The food frequency data were transformed into a score of annual consumption. The DP were theoretically derived using principal component analysis. It was identified four DP, as follows: Healthy (with high loadings for fruits, vegetables and wholegrains), Brazilian traditional (with high loadings for foods consumed daily by Brazilians such as rice, beans, pasta, potatoes and red meat), sugar and carbohydrates (with high loadings for sugar, cookies, cakes, soft drinks, chocolate and bread) and fast-food (with high loadings for ultra-processed foods). The four DP together explained 27·34 % of the total variance. Each DP score factor was included in the analyses as a continuous variable.

### Obesity and abdominal obesity: outcomes

The study outcomes were obesity (no/yes) and abdominal obesity (no/yes). Obesity was defined as having a BMI of ≥30 kg/m², calculated by dividing body weight (kg) by height (m) squared, and classified using cut-off points defined by WHO indicating the nutritional status of individuals. Two measurements of weight (kg) and height (m) were made, and the mean was used to calculate an individual’s BMI. A calibrated electronic balance (Marte®, model PP 200) was used to weigh out participants, without shoes and with as few accessories and clothes as possible. Participants stood upright in the centre of the balance, distributing their weight equally between both feet. A portable stadiometer (Secca®, model 213) was used to measure participants’ height. Measurements were made without shoes and accessories on the head, with the individual positioned so that their calf, buttocks, shoulders and head touched the vertical surface of the instrument wherever possible. Facing forward, as in the Frankfurt Plan, the support was positioned over the head so that it only pressed the hair. The measurement was recorded immediately^(27,28)^. Waist circumference was measured in centimetres at the midpoint between the iliac crest and the lowest rib. This procedure was done twice, and the mean value between the two measurements was used. For analysis purposes, abdominal obesity is ≥ 88 cm for women and ≥ 102 cm for men^(27)^.

### Confounders

Demographic and socio-economic variables included were gender (self-reported woman/man), age (reported in complete years) skin colour/race/ethnicity (self-reported according to the categories proposed by the Brazilian Institute of Geography and Statistic (IBGE): White/Black/Brown/Yellow/Indigenous), education (incomplete elementary school/complete elementary school/complete high school/complete higher (university) education), monthly family income (minimum wage (MW): <MW/1–2 times the MW/2–3 times the MW/3–4 times the MW/4–5 times the MW/>5 times the MW). The MW in 2019 in Brazil is R$ 998,00 (∼ $200).

### Statistical analyses

Data entry was performed in the EpiData program, with double entry and subsequent comparison. Data analyses were performed using Stata Version 14 (StataCorp) and Mplus version 8.2. Descriptive statistics were used to characterise the study sample and describe the experience of racial discrimination, DP, obesity, and abdominal obesity according to demographic and socio-economic characteristics. Categorical variables were described using measures of absolute (*n*) and relative (%) frequency, while numerical variables were described using measures of mean and standard deviation, and Pearson’s *χ* ^2^, Student’s *t* test, or ANOVA were used to assess associations. All analyses considered a *P*-value less than 5 % (*P* < 0·05) as statistically significant.

Equation modeling was performed to evaluate the relationships between variables. This is performed employing simultaneous confirmatory factor analysis and regression analysis; thus, the model consisted of two parts: a measurement model and a path model.

The path model was primarily designed to test the study’s hypotheses and was based on a priori theory. A directed acyclic graph was created to illustrate assumptions about the causal relationships among variables and potential confounding variables (Dagitty version 3.2; Johannes Textor, Radboud University Nijmegen) (see online supplementary material, Supplementary Figure1). There is a direct effect of the experience of discrimination on DP and also on obesity and abdominal obesity controlling for socio-economic characteristics. DP, our mediator variables, are determined by the experience of discrimination and determines both obesity and abdominal obesity, controlling for socio-economic characteristics. The total effects also were measured for each model. We built four path models, one for each DP. Covariables associated with the EOD in bivariate analysis were included in the models. Also, gender was included because it is an important factor associated with racial discrimination and obesity in the literature. Colour/race was not included in the models as an adjusted covariate because it is an indirect measure of racial discrimination and is highly correlated with racial discrimination experiences, which is a direct measure of racial discrimination^(29)^. Thus all analyses are adjusted by age, gender, schooling, income and living area.

Models were estimated using weighted least squares mean and variance adjusted estimator. Items that had moderate factor loadings (>=0·6) were retained^(30)^. The fit of both measurement and path models was assessed by means of fit indices, including the *χ* ^2^ (*P*-value > 0·05), the comparative fit index (CFI > 0·95), the Tucker–Lewis index (TLI > 0·95), the fit index root mean square error of approximation (RMSEA =< 0·06) and the standardised root mean squared residual (SRMR < 0·08)^(31)^. The post-estimation command ‘MODINDICES’ was used to improve the fit of the models by adding theoretically sound correlations among error terms. Standardised coefficients are present in the results.

## Results

### Sample description and bivariable analyses

A total of 400 participants with a mean age of 47·2 years (s d = 13·9 years) were included in this study. Most of the sample was composed of women (75 %) and self-reported White colour (62·3 %). Regarding education, 39·9 % had 11 years of education, while 48·4 % of the sample reported a family income of 3–5 MW. Higher means of racial discrimination were observed among younger people, who self-reported Black colour, those living in lower-income areas and completed high school education. Higher scores of healthy DP were observed among women, older people, White people, those who live in higher-income areas, and those who have higher levels of income and education. Higher scores of the Brazilian traditional DP were observed among younger and Brown people, those who live in lower-income areas and have lower levels of income and education. Greater scores of the sugar and carbohydrates DP were found among younger and Black people, those who live in lower-income areas and those have lower levels of income and education. Higher scores of the fast-food DP were observed among younger and White people, those who live in higher-income areas and those have higher levels of education and income. Obesity was associated only with lower education levels, while abdominal obesity was associated with being a woman, being older and having lower levels of education (Table 1).

**Table 1:** Experience of discrimination, DP and obesity according to demographic and socio-economic characteristics in Brazilian adults

			Experience of discrimination		Healthy DP		Traditional DP		Sugar and CHO DP		Fast-food DP		Obesity*		Abdominal obesity†	
Variables	*n*	%	Mean	sd	Mean	sd	Mean	sd	Mean	sd	Mean	sd	*n*	%	*n*	%
Gender			*P* = 0·692		*P* = 0·015		*P* = 0·007		*P* = 0·061		*P* = 0·222		*P* = 0·381		*P* < 0·001	
Women	300	75·00	2·29	5·36	−0·07	1·02	−0·07	0·97	−0·05	0·93	−0·03	0·99	104	34·07	164	54·67
Men	100	25·00	2·54	5·32	0·20	0·90	0·23	1·04	0·16	0·93	0·10	0·99	30	30·00	31	31·00
Age (years)			*P* = 0·007		*P* = 0·006		*P* = 0·049		*P* < 0·001		*P* < 0·001 ‡		*P* = 0·060		*P* < 0·001 ‡	
18–33	91	22·75	3·45	6·69	−2·26	1·07	0·18	1·06	0·20	1·07	0·30	1·30	22	24·18	29	31·87
34–45	79	19·75	2·92	5·59	−0·09	1·01	0·08	0·95	0·24	1·20	0·02	0·90	35	44·30	40	50·63
46–54	80	20·00	1·88	5·05	−0·02	0·94	0·06	0·87	−0·12	0·80	0·01	0·93	27	33·75	40	50·00
55–60	71	14·75	1·36	3·59	0·22	0·92	−0·23	1·04	0·00	0·94	−0·19	0·85	20	33·75	38	53·52
61–70	79	19·75	1·90	7·78	0·21	0·95	−0·14	1·02	−0·35	0·80	−0·20	0·79	30	37·97	48	60·76
Living area			*P* < 0·001		*P* < 0·001		*P* < 0·001		*P* < 0·001		*P* = 0·016		*P* = 0·216		*P* = 0·920	
Lower income	201	50·25	3·29	6·21	−0·20	0·99	0·39	1·10	0·36	1·09	−0·12	1·16	(3	36·32	98	49·25
Higher income	199	49·75	1·41	4·11	0·21	0·96	−0·40	0·68	−0·36	0·74	0·12	0·78	61	30·65	97	48·74
Race/skin colour			*P* < 0·001		*P* = 0·017		*P* < 0·001		*P* < 0·001		*P* = 0·065		*P* = 0·143		*P* = 0·467	
White	250	62·50	0·51	2·50	0·10	0·97	−0·18	0·93	−0·15	0·88	0·08	0·88	75	30·0	118	47·39
Black	78	19·50	7·50	7·97	−0·10	1·12	0·35	1·11	0·44	1·24	−0·09	1·43	31	40·26	37	48·05
Brown	72	18·00	3·16	5·32	−0·24	0·91	0·26	0·94	0·05	0·94	−0·20	0·78	28	38·89	40	55·56
Education			*P* = 0·028		*P* = 0·08		*P* < 0·001‡		*P* < 0·001‡		*P* < 0·001‡		*P* = 0·039‡		*P* = 0·025‡	
ES incomplete	78	19·50	2·85	5·71	−0·04	1·10	0·48	1·27	0·38	1·25	−0·23	1·23	31	40·26	43	55·84
ES complete	73	18·25	2·53	5·68	−0·11	1·13	0·23	0·81	0·17	0·96	−0·21	0·85	27	36·99	39	54·17
HS complete	146	36·50	2·94	5·85	−0·05	0·90	−0·07	0·92	−0·01	0·98	0·01	0·98	51	34·93	70	47·95
UE complete	103	25·75	1·01	3·64	0·19	0·94	−0·43	0·75	−0·40	0·62	0·31	0·83	25	24·27	43	41·75
Income (MW)			*P* = 0·058 ‡		*P* < 0·001 ‡		*P* < 0·001‡		*P* < 0·001‡		*P* < 0·001‡		*P* = 0·074‡		*P* = 0·105	
<1	22	5·51	3·00	4·15	−0·38	0·77	0·61	1·08	0·40	0·97	−0·34	0·83	8	38·10	11	52·38
1–2	112	28·10	2·97	6·34	−0·17	1·04	0·28	1·05	0·10	0·97	−0·25	1·00	42	37·50	60	53·57
3–5	193	48·40	2·27	5·10	0·03	1·02	−0·10	0·97	0·02	1·06	0·06	1·07	67	34·72	98	51·04
>5	72	18·00	1·45	4·57	0·27	0·85	−0·32	0·74	−0·34	079	0·31	0·70	17	23·61	26	36·11

DP, dietary pattern; CHO, carbohydrates; ES, elementary school; HS, high school; UE, university education; MW, minimum wage; *P*, *P*-value for Pearson’s *χ* ^2^ for categorical variables or *t* test/ANOVA for continuous variables.

* Sample size = 399.

† Sample size = 398.

‡ *P*-value for linear trend.

### Confirmatory factor analyses

First, we stated the analysis confirming the factor structure of the nine discrimination items of EOD scale. Post-estimation commands suggested that the model fit could significantly be improved by dropping out the variable ‘getting housing’, one of the specified situations of discrimination. After this, the measurement model presented a good fit for all fit indices: *X* ^2^(2) = 24·87, *P* = 0·206; RMSEA = 0·025; CFI = 0·998; TLI = 0·997; and SRMR = 0·044 (see online supplementary material, Supplementary Table 1). This analysis was conducted with 398 individuals who had complete data in all variables.

### Relationship between racial discrimination, dietary patterns and obesity

**Fig. 1:**
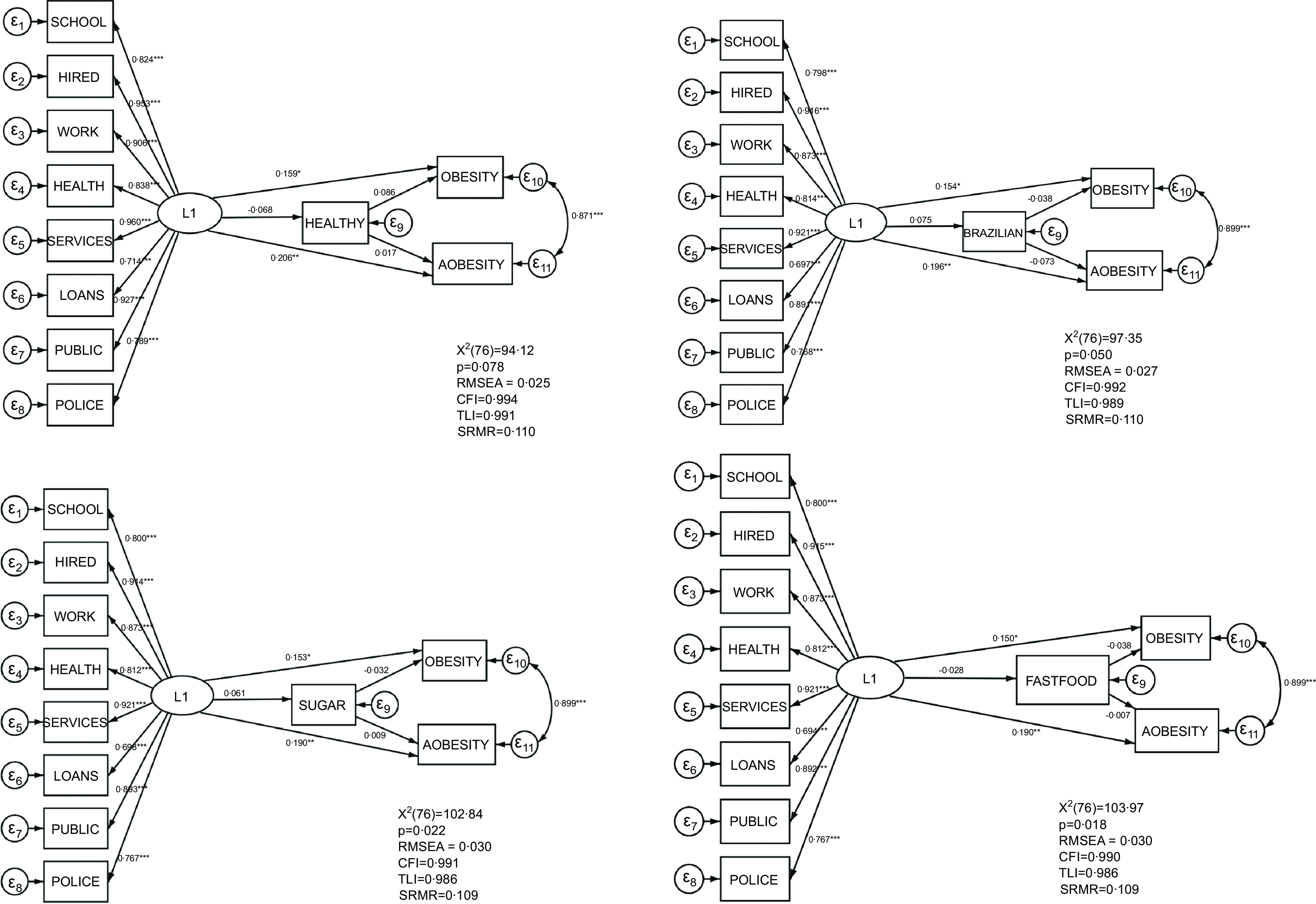
Structural equation modelling of experience of racial discrimination (L1), dietary patterns (healthy, Brazilian, sugar and carbs and fast-food), obesity and abdominal obesity. All estimates are standardised. *P < 0·05, **P < 0·01, ***P < 0·00. All analyses are adjusted by age, gender, schooling, income and living area. RMSEA, root mean square error of approximation; CFI, comparative fit index; TLI, Tucker–Lewis index; SRMR, standardised root mean squared residual.

The models of the direct and indirect association between experience of racial discrimination with each DP and obesity and abdominal obesity are presented in Fig. 1. All the models presented a modest fit. The *χ* ^2^ presented *P* < 0,05 in two models (sugar and carbohydrates DP and fast-food DP) and the SRMR, which was >0·08 in all models. RSMEA, CFI and TLI were good in all models. Modification indices did not suggest any significant improvement in the model’s fit^(31)^.

Across all models, experiencing racial discrimination led to an increase in obesity (healthy DP model: *β* = 0·153, *P* < 0·05; Brazilian DP model: *β* = 0·156, *P* < 0·05; sugar and carbohydrates DP model: *β* = 0·156, *P* < 0·05; and fast-food DP: *β* = 0·153, *P* < 0·05) and abdominal obesity (healthy DP model: *β* = 0·206, *P* < 0·01; Brazilian DP model: *β* = 0·210, *P* < 0·01; sugar and carbohydrates D: *β* = 0·204, *P* < 0·01; and fast-food DP: *β* = 0·204, *P* < 0·01). The total effects were also positively significant for both outcomes for all models and had values similar to the direct effects (Fig. 1 and Table 2).

**Table 2:** Direct, indirect and total standardised effects of experience of racial discrimination on obesity and abdominal obesity through DP

Experience of racial discrimination	Obesity *β*	se	Abdominal obesity *β*	se
Healthy DP				
Direct effect	0·159*	0·072	0·206**	0·078
Indirect effect	−0·006	0·007	−0·001	0·005
Total effect	0·153*	0·072	0·204**	0·078
Brazilian DP				
Direct effect	0·156*	0·072	0·210**	0·079
Indirect effect	−0·003	0·006	−0·006	0·007
Total effect	0·153*	0·072	0·205**	0·078
Sugar and CHO DP				
Direct effect	0·156*	0·073	0·204*	0·078
Indirect effect	−0·002	0·005	0·001	0·004
Total effect	0·154*	0·072	0·205**	0·078
Fast-food DP				
Direct effect	0·153*	0·072	0·204**	0·078
Indirect effect	0·001	0·003	0·000	0·002
Total effect	0·154*	0·072	0·205**	0·078

DP, dietary patterns; CHO, carbohydrates.

* *P* < 0·05.

** *P* < 0·01.

The experience of racial discrimination did not show direct effects on any DP (H2 and H3), and racial discrimination did not have an indirect effect on obesity nor on abdominal obesity via DP (H4 and H5). Thus, experienced racial discrimination was positively associated with obesity and abdominal obesity independently of the mediation of food consumption (Fig. 1 and Table 2). The standardised coefficients of the covariables in the four models are available (see online supplementary material, Supplementary Table 2.

## Discussion

Our results suggest that higher levels of self-reported experiences of racial discrimination were related to obesity and abdominal obesity, and these associations were independent of healthy or unhealthy DP among Brazilian adults. Thus, it confirms only our first hypothesis (H1: Experience of racial discrimination has a positive direct effect on obesity and abdominal obesity).

Previous longitudinal studies found results in this way for obesity and waist circumference^(12,13)^. In Brazil, a study investigated the association between perceived racial discrimination and obesity incidence in a 4-year follow-up of the Brazilian Longitudinal Study of Adult Health (*ELSA-Brasil*) and demonstrated that obesity incidence was higher among Black individuals reporting racial discrimination than Black individuals who did not report this experience^(20)^.

Racially discriminatory events may trigger physical and psychological stress responses. The neuroendocrine stress response is a possible physiological explanation for weight gain and fat accumulation without food consumption alterations. Chronic stress leads to activation of the hypothalamus–pituitary–adrenal axis, which affects health outcomes. Activation of the hypothalamus–pituitary–adrenal axis causes an inflammatory process mediated by glucocorticoid hormones (cortisol and corticosterone). Excess cortisol concentrations have been associated with visceral fat accumulation, since cortisol activates lipoprotein lipase, increasing fat retention, mainly in the abdominal region due to the high density of receptors in intra-abdominal adipose tissue^(17)^. In addition, glucocorticoids are associated with visceral fat through their effect on lipid metabolism. In the presence of insulin, increased cortisol concentrations inhibit lipid mobilisation and increase lipid accumulation, either directly by stimulation of lipoprotein lipase or indirectly by inhibiting the lipolytic effects of growth hormone^(18)^.

The hypotheses that experience of racial discrimination was directly associated with DP (H2 and H3) and that the association between experience of racial discrimination and obesity was mediated by DP (H4 and H5) were not confirmed in this study. However, previous epidemiological studies have demonstrated an association between experience of racial discrimination and unhealthy diets^(10)^. It has been postulated that chronic activation of the hypothalamus–pituitary–adrenal axis, which alters glucose metabolism, promotes insulin resistance and affects several appetite-related hormones and feeding neuropeptides. It causes changes in the mechanisms of hunger and satiety, enhancing the propensity to eat high-calorie palatable foods. It is also suggested that in stressful situations, such as experiences of discrimination, individuals appeal to coping strategies to alleviate stress, such as the consumption of high-calorie and palatable foods^(32)^.

These theories have been linking stress to increased consumption of high-fat and sugar foods and high-caloric and palatable foods. In this study, it was identified two DP composed by high-caloric and palatable foods, the sugar and carbohydrates DP and the fast-food DP, which may have diluted the effect of the association between the consumption of these types of food and the stress due to experiences of racial discrimination. The present study is the first in this field to use the DP approach. We measured the total food consumption, in the last year, and identified DP through *a posteriori* statistical method. This approach evaluates the total diet and emerged as an alternative method of measuring dietary exposures since people eat meals that include many foods and nutrients. Hence, studies that are reduced to just one nutrient, food or food group are not able to account for how these elements may act in combination. DP, identified through last year’s food consumption, also has been described as a better approach to understanding the relationship between diet and long latency outcomes, such as obesity^(33)^, and also can be better to evaluate associations to life course experiences of racial discrimination. However, as *a posteriori* method, DP varies between populations, and further studies with this approach should be carried out to deeply explore its association with racial discrimination.

Experiences of racism are not enough to measure the impact of racism on health, though. On a broader level, structural and institutional racial discrimination has several consequences on health and nutritional outcomes. As a political determinant of health, racism encompasses the environmental, cultural and socio-economic conditions people are born and live. Legal rules and governmental policies related to nutrition regulations and food options, food environment, healthcare access, acculturation of food practices among discriminated social groups, and targeted marketing of unhealthy foods and commodities may cause and reinforce unhealthy DP and bad health outcomes. For example, there is evidence that the food environment is important for understanding how racial discrimination affects an individual’s eating behaviour. In Black neighbourhoods is less frequent the presence of establishments that sell fresh or minimally processed food with sufficient quantity and adequate quality^(34)^. Thus, major efforts must be made in the future to establish new measures of structural determinants of health to reveal the privilege or prejudice of different social groups and challenge biological essentialism that pervades scientific studies on health and nutrition^(6)^.

Additionally, Porto Alegre, with approximately 1·30 million inhabitants, has about 25 % of its residents identifying as Black or Brown – a rate lower than the national average of 56 % for the Brazilian population. However, it stands out as the Brazilian capital with the highest rates of social inequalities between the White and Black/Brown populations. Previous studies conducted with the same sample unveiled racial disparities in health within this population. The neighbourhoods with the highest percentage of Black residents were associated with lower income and exhibited the poorest food environments, marked by a limited availability of healthy foods^(33)^. Moreover, individuals identifying as Black and Brown were found to be more likely to experience overweight^(35)^. Thus, addressing these social and health disparities in Brazilian Black and Brown populations requires continuous research to monitor and understand the evolving dynamics of social and health disparities. This, in turn, will contribute to the development of evidence-based interventions and policies.

### Limitations and strengths

It is a cross-sectional study and therefore has limitations in determining causality. Furthermore, cross-sectional studies are unable to determine the temporal relationship between exposure variables and outcomes. Individuals with obesity experience discrimination in many settings, and the associations between racism and weight discrimination could be interrelated. Regarding DP, the use of a retrospective method to measure food intake (FFQ) may lead to recall errors, and it is also important to highlight that the statistical method used to assess DP is limited by the subjectivity of the decisions taken by the researchers. In addition, the FFQ collected only the frequency of food consumption and prevented the analysis from taking into account the amount of intake.

The confirmatory factor analysis performed to confirm the structural validity of the discrimination construct of EOD scale showed a very good fit. The model achieved a better fit with the exclusion of the variable ‘getting housing’, one of the specified situations of experience of discrimination. The study that validated the scale in Brazil also found low factor loadings for this item, and the authors highlighted that the assessment of discrimination in getting housing might be more pertinent in the USA, where the segregation patterns of Black people continue to be the highest among all ethnic-racial groups, while the same intensity of segregation may not be observed in Brazil^(26)^. On the other hand, adjustment indices *χ* ^2^ and SRMR were not ideal in the final path models. It has been noted that because *χ* ^2^ statistic is, in essence, a statistical significance test, it is sensitive to sample size. In addition, SRMR will be lower when there is a high number of parameters in the model, which may not the case of our parsimonious models^(36)^.

This study was innovative in at least three aspects: (1) the understanding of the role of food consumption in the relationship between perceived racial discrimination and obesity and abdominal obesity; (2) studied the association between the experience of racial discrimination and food consumption in low- and middle-income countries; and (3) applied DP approach in the research field of racial discrimination.

### Conclusion

In this cross-sectional study, the self-report experience of racial discrimination was directly related to obesity and abdominal obesity, and its relationship was independent of the consumption of healthy or unhealthy DP. Future research employing a longitudinal design across diverse populations is essential to confirm these findings. Health and nutrition researchers need to consider more seriously the impact of racism and racial discrimination on nutritional outcomes in their studies. It is crucial to deeply understand the impact of all forms of racism on food and nutrition outcomes in the lifetime and propose food policies that take into account the health inequities caused by racism^(37)^. It is especially important in countries scared by racism, such as Brazil.

## Supporting information

Fanton et al. supplementary material Fanton et al. supplementary material

## Supplementary material

For supplementary material accompanying this paper visit https://doi.org/10.1017/S1368980024000338
