# Supplementary material for: Direct and indirect associations of experience of racial discrimination, dietary patterns and obesity in adults from southern Brazil
Source: Public Health Nutr. 2024 Feb 1;27(1):e60. doi: 10.1017/S1368980024000338 (PMC10897576; doi:10.1017/S1368980024000338)
Supplement: Fanton et al. supplementary material [file S1368980024000338sup001.pdf]

SUPPLEMENTARY MATERIAL

Supplementary Figure 1. Directed acyclic graph (DAG) of the causal relationships between racial discrimation, dietary patterns and obesity/abdominal obesity, and potential confounding variables.

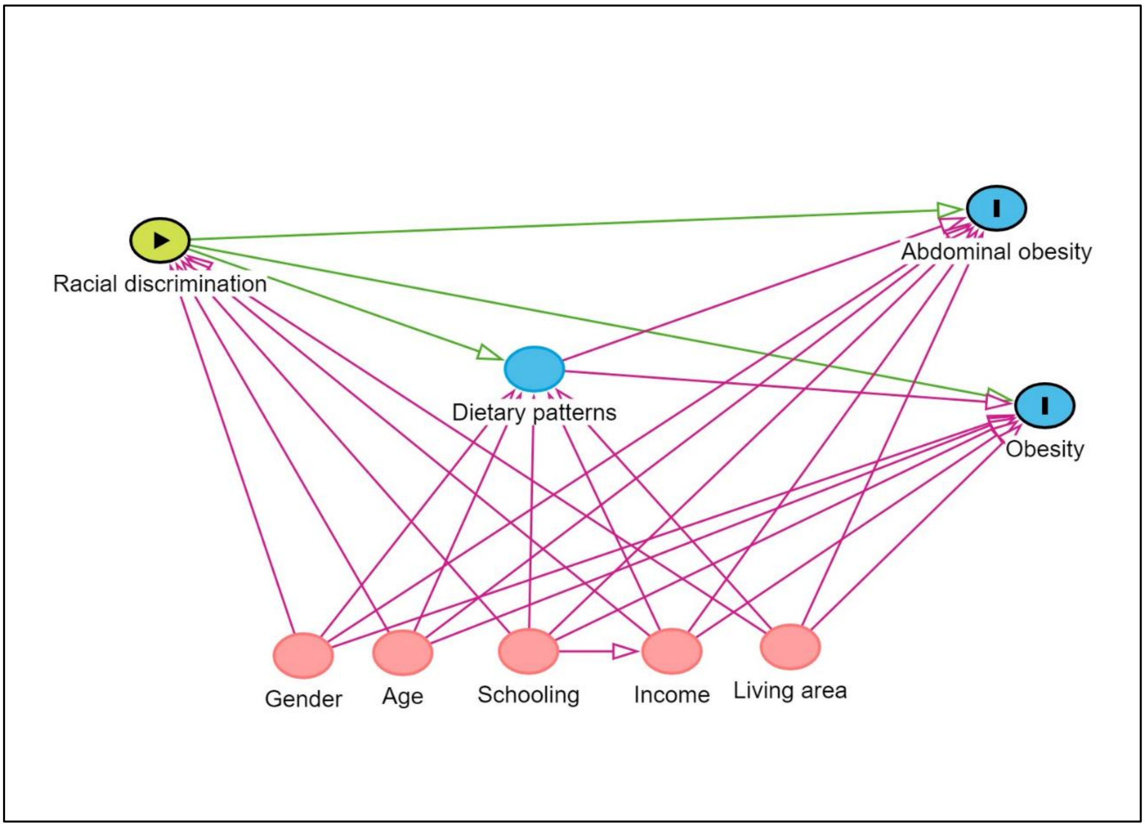

Supplementary Table 1. Confirmatory factor analysis standardized estimations of Experiences of Discrimination scale (OED).

OED questions	Standardized coefficients	SE	p-value
School	0.785	0.045	<0.001
Hired	0.893	0.027	<0.001
Work	0.871	0.033	<0.001
Health	0.796	0.053	<0.001
Service	0.918	0.027	<0.001
Loans	0.685	0.081	<0.001
Public setting	0.878	0.034	<0.001
Police	0.702	0.066	<0.001
Model fit information			
Chi-Square	4.872 (p=0.206)		
RMSEA	0.025		
CFI	0.998		
TLI	0.997		
SRMR	0.044		

SE= standard error

Supplementary Table 2. Standardized coefficients (**β**) of covariables included in the four path models.

	β (SE)			
Discrimination on				
Gender	-0.162 (0.159)	-	-	-
Age	-0.006 (0.006)	-	-	-
Schooling	-0.040 (0.086)	-	-	-
Income	-0.061 (0.113)	-	-	-
Living area	-0.442 (0.174)*	-	-	-
Dietary Pattern on	***Healthy***	***Traditional***	***CHO and Sugars***	***Fast-food***
Gender	0.324 (0.120)*	-0.348(0.103)*	-0.252 (0.103)*	-0.109 (0.116)
Age	0.010 (0.004)*	-0.006 (0.003)*	-0.008 (0.004)*	-0.013 (0.004)**
Schooling	-0.052 (0.059)	-0.146 (0.048)*	-0.125 (0.056)*	0.082 (0.056)
Income	0.170 (0.080)*	-0.094 (0.069)	0.009 (0.071)	0.169 (0.082)*
Living area	0.273 (0.174)	-0.496 (0.119)*	-0.517 (0.127)**	0.089 (0.132)
Obesity on				
Dietary pattern	0.086 (0.067)	-0.039 (0.073)	-0.032 (0.067)	- 0.038 (0.066)
Gender	0.103 (0.157)	0.117 (0.158)	0.122 (0.155)	0.126 (0.155)
Age	0.005 (0.005)	0.005 (0.005)	0.005 (0.005)	0.005 (0.005)
Schooling	-0.092 (0.078)	-0.102 (0.079)	-0.101 (0.079)	-0.094 (0.079)
Income	0.068 (0.099)	-0.057 (0.099)	-0.053 (0.099)	-0.047 (0.099)
Living area	0.007 (0.171)	0.011 (0.176)	0.014 (0.174)	0.034 (0.171)
Abdominal Obesity on				
Dietary pattern	0.017 (0.067)	-0.079 (0.069)	0.010 (0.066)	-0.007 (0.065)
Gender	0.648 (0.156)*	0.117 (0.158)	0.656 (0.156)**	0.653 (0.155)**
Age	0.018 (0.005)*	0.005 (0.005)	0.018 (0.005)**	0.018 (0.005)**
Schooling	-0.080 (0.078)	-0.102 (0.079)	-0.080 (0.079)	-0.081 (0.078)
Income	-0.085 (0.097)	-0.057 (0.099)	-0.082 (0.097)	-0.080 (0.097)
Living area	0.147 (0.173)	0.011 (0.176)	0.157 (0.157)	0.153(0.172)

CHO=Carbohydrates; SE=standard error; * P-value <0.05; ** P-value <0.001
